# Prognostic value of the C-reactive protein/Albumin Ratio (CAR) in patients with operable soft tissue sarcoma

**DOI:** 10.18632/oncotarget.20990

**Published:** 2017-09-18

**Authors:** Yao Liang, Wei Xiao, Yuan-Xiang Guan, Wei Wang, Huo Ying Chen, Cheng Fang, Xing Zhang, Zhi-Wei Zhou

**Affiliations:** ^1^ Sun Yat-Sen University Cancer Center, State Key Laboratory of Oncology in South China, Collaborative Innovation Center for Cancer Medicine, Guangzhou 510060, People's Republic of China

**Keywords:** C-reactive protein, albumin, inflammation-based prognostic factors, soft tissue sarcoma, prognosis

## Abstract

**Background:**

The preoperative C-reactive protein/Albumin ratio (CAR) is valuable for predicting the prognosis of patients with various types of cancers. The aim of the present study is to investigate the prognostic value of the preoperative CAR and compare it with other systemic inflammatory response markers in patients with soft tissue sarcoma (STS).

**Methods:**

This retrospective study included 206 patients with STS. The optimal cutoff value of the CAR was determined by receiver operating characteristic (ROC) analysis. The impact of the CAR and other clinicopathological features on overall survival (OS) and disease-free survival (DFS) was evaluated using univariate and multivariate Cox regression analyses. Kaplan-Meier survival analyses were used to compare groups classified by the CAR. Additionally, the area under the receiver operating characteristic curve (AUC) was used to compare the predictive ability of the CAR, high-sensitivity modified Glasgow prognostic score (Hs-mGPS), neutrophil-lymphocyte ratio (NLR) and platelet-lymphocyte ratio (PLR).

**Results:**

The optimal cut-off value of the CAR was 0.1035 according to the ROC analysis. An increased CAR (≥0.1035) was significantly associated with older age, larger tumor size, deep tumor location, higher tumor grade and more advanced American Joint Committee on Cancer (AJCC) stage (all P<0.05). Patients with an elevated CAR (≥0.1035) exhibited a shorter median survival time and lower 5-year OS rate than those with a CAR<0.1035 (68.2 vs 115.8 months, P = 0.000; 44.6% vs 80.9%, P = 0.000, respectively). The results of a multivariate analysis indicated that the CAR (Hazard ratio (HR) 2.47, 95% confidence interval (CI) 1.47-4.14, P = 0.001) was an independent prognostic factor for OS along with tumor grade (P<0.05). Additionally, the CAR exhibited a greater AUC value (0.662) than the NLR and PLR, but the value was equal to the Hs-mGPS.

**Conclusions:**

The preoperative CAR is an independent prognostic factor predicting prognosis in STS and exhibits superior prognostic ability compared to the established inflammation-based prognostic indices.

## INTRODUCTION

Soft tissue sarcomas (STSs) arising from almost any embryonic mesodermal tissue, are a group of rare tumors that comprise approximately 1-2% of malignancies in adults and encompass greater than 50 different subtypes [1, 2]. Despite improvements in local control rates with extensive local resections and radiation therapy, STS patients, with high-grade tumors are at a particularly significant risk of recurrence and distant metastasis after multimodality treatment. Greater than 30% of patients with STS will develop metastases and exhibited estimated 5-year survival rates of approximately 50%[3–5]. Therefore, it is important to identify easily obtainable, widely applicable and reliable prognostic factors that, might help guide treatment options and improve the risk stratification ability.

Increasing investigations have indicated that inflammation plays an important role in cancer development and progression [6]. In recent years, the prognostic value of various inflammation-based prognostic factors derived from routine tests, such as the neutrophil-lymphocyte ratio (NLR), platelet-lymphocyte ratio (PLR), Glasgow Prognostic Score (GPS), modified Glasgow prognostic score (mGPS), and high-sensitivity modified Glasgow prognostic score (Hs-mGPS), have been validated in numerous cancer types [7, 8].

Notably, preoperative C-reactive protein (CRP), which represents a marker of systemic inflammatory reactions, has been widely reported as a prognostic factor for poor survival in patients with various cancer types, including STS [9, 10]. Additionally, various studies have demonstrated that the preoperative nutritional status, such as anemia, hypoalbuminemia, weight loss and low body mass index (BMI), are associated with a low survival in STS patients [11]. Recently, a novel prognostic index, namely, the preoperative CRP/Albumin ratio (CAR) in combination with systemic inflammation and nutritional status, has been reported as an independent prognostic marker in small-cell lung cancer [12], pancreatic cancer [13], esophageal cancer [14] and hepatocellular carcinoma [15]. However, according to current knowledge, no study has been previously published with a particular focus on the prognostic value of the CAR in STS patients. Therefore, the objective of the present study was to evaluate the prognostic impact of the preoperative CAR on disease-free survival (DFS) and overall survival (OS) in STS patients and to examine any links between an increased CAR and clinical characteristics.

## RESULTS

### Patient and tumor characteristics

A total of 206 subjects were selected, with a median age of 39 years (range:10-78 years). Among these patients, 117 (56.8%) were males, 89 (43.2%) were females, 63 (30.6%) died, and 80 (38.8%) relapsed at the last follow-up.

Preoperative blood sample analyses revealed that the median CRP value was 1.825 mg/L (range: 0.4-118.49 mg/L), and the median Albumin(Alb) value was 42.95 g/L (range: 26.40-57.70 g/L). The Hs-mGPS score was 0 in 123 cases (59.7%), 1 in 73 cases (35.4%) and 2 in 10 cases (4.9%). The tumor's pathological subtypes included so-called MFH in 56 patients (27.2%), fibrosarcoma in 38 (18.4%), synovial sarcoma in 25 (12.1%), and liposarcoma in 22 (10.7%). In total, 49 patients were histologically classified as having grade one sarcomas, 76 patients had grade two, and 67 patients had grade three. Overall, 49 (23.8%) patients were classified as stage I, 94 (45.6%) as stage II, and 49 (23.8%) as stage III or stage IV. The baseline characteristics of the 206 patients are presented in Table 1.

**Table 1:** Baseline characteristics of all patients (N=206)

	Number of patients (%)
Sex	
male	117(56.8)
female	89(43.2)
Age (years)	
<50	136(66)
≥50	70(34)
ECOG Performance status	
0	159(77.2)
≥1	47(22.8)
Hs-mGPS	
0	123(59.7)
1	73(35.4)
2	10(4.9)
Pathological types	
Fibrosarcoma	38(18.4)
Liposarcoma	22(10.7)
Undifferentiated pleomorphic sarcoma/MFH	56(27.2)
Leiomyosarcoma	9(4.4)
Synovial sarcoma	25(12.1)
Rhabdomyosarcoma	13(6.3)
Alveolar soft part sarcoma	8(3.9)
Angiosarcoma	6(2.9)
Malignant peripheral nerve sheath tumor	8(3.9)
Mesenchymal chondrosarcoma	7(3.4)
Others	14(6.8)
Tumor size	
<5cm	101(49)
≥5cm	105(51)
Tumor site	
Upper extremity	25(12.1)
Lower extremity	62(30.1)
Thoracic/trunk	55(26.7)
Intra-abdominal	27(13.1)
Head/neck	33(16)
Others	4(1.9)
Tumor depth	
Superficial	92(44.7)
Deep	114(55.3)
Tumor grade	
G1	49(23.8)
G2	76(36.4)
G3	67(32.5)
Missing	15(7.3)
AJCC stage	
IA+IB	49(23.8)
IIA+IIB	94(45.6)
III+IV	49(23.8)
Missing	14(6.8)
NLR	
<1.64	78(37.9)
≥1.64	128(62.1)
PLR	
<151.9	153(74.3)
≥151.9	53(25.7)
CAR	
<0.1035	141(68.4)
≥0.1035	65(31.6)
Recurrence	
YES	80(38.8)
NO	126(61.2)
Metastasis	
YES	47(22.8)
NO	159(77.2)
End-point	
Alive	143(69.4)
Dead	63(30.6)

N = number, Hs-mGPS = high sensitivity modified Glasgow prognostic score, AJCC = American Joint Committee on Cancer, NLR = neutrophil-lymphocyte ratio, PLR = platelet-lymphocyte ratio, CAR = C-reactive protein-Albumin ratio.

### The relationship of serum CRP and Alb with OS

We explored the association of the serum CRP and Alb with OS. The results revealed a significant negative correlation between serum CRP level and OS (r = −0.212, P = 0.002) (Additional file 1: Supplementary Figure 1A) and a significant positive correlation between serum Alb level and OS (r = 0.232, P = 0.001) (Additional file 1: Supplementary Figure 1B).

### Determination of the optimal cut-off value

**Figure 1:**
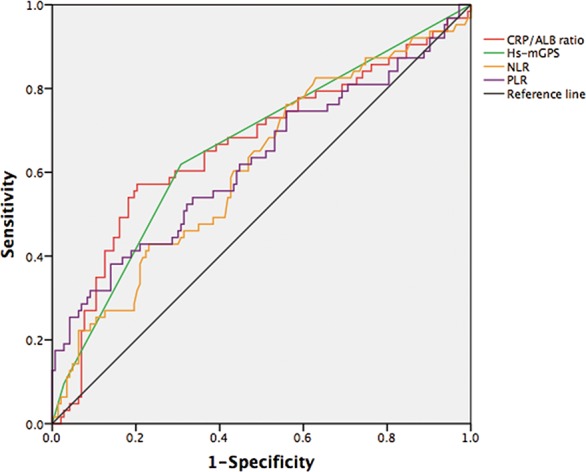
ROC curve analyses for inflammation-based factors

After performing a receiver operating characteristic (ROC) analysis and calculating the areas under the curve (AUC) (Figure 1 and Additional file 2: Supplementary Table 1), the optimal cutoff value for the CAR was 0.1035 (AUC: 0.662, 95% confidence intervals (CI): 0.577-0.748). The optimal cutoff for the NLR was 1.64 (AUC: 0.612, 95% CI: 0.527-0.697), and the optimal cutoff for the PLR was 151.9 (AUC: 0.624, 95% CI: 0.535-0.713). Based on this result, all patients were categorized into the high-CAR (CAR≥0.1035, n = 65, 31.6%) and low-CAR (CAR<0.1035, n = 141, 68.4%) groups.

### Correlation between preoperative CAR and clinicopathological characteristics

Table 2 presents the associations between the CAR and other variables. An elevated CAR was significantly associated with age (P = 0.002), tumor diameter (P = 0.000), tumor depth (P = 0.002), tumor grade (P = 0.001), American Joint Committee on Cancer (AJCC) stage (P = 0.001) and other inflammatory markers (NLR, PLR), whereas no association with gender was noted (P = 0.217).

**Table 2:** Correlation of the CAR with the clinicopathological characteristics

	CAR		*p* value
	<0.1035, N (%)	≥0.1035, N (%)	
Gender			
male	76(53.9)	41(63.08)	0.217
female	65(46.1)	24(36.92)	
Age (years)			
<50	103(73.05)	33(50.77)	0.002
≥50	38(26.95)	32(49.23)	
Tumor diameter			
<5cm	83(58.87)	18(27.69)	0.000
≥5cm	58(41.13)	47(72.31)	
Tumor site			
Upper extremity	19(13.48)	6(9.23)	0.001
Lower extremity	48(34.04)	14(21.54)	
Thoracic/trunk	43(30.5)	12(18.46)	
Intra-abdominal	9(6.38)	18(27.69)	
Head/neck	19(13.48)	14(21.54)	
Others	3(2.13)	1(1.54)	
Tumor depth			
Superficial	73(51.77)	19(29.23)	0.002
Deep	68(48.23)	46(70.77)	
Tumor grade			
G1	44(31.21)	5(7.69)	0.001
G2	47(33.33)	28(43.08)	
G3	38(26.95)	29(44.62)	
Missing	12(8.51)	3(4.62)	
AJCC stage			
IA+IB	43(30.5)	6(9.23)	0.001
IIA+IIB	60(42.55)	34(52.31)	
III+IV	26(18.44)	23(35.38)	
Missing	12(8.51)	2(3.08)	
NLR			
<1.64	66(46.81)	12(18.46)	0.000
≥1.64	75(53.19)	53(81.54)	
PLR			
<151.9	111(78.72)	42(64.62)	0.031
≥151.9	30(21.28)	23(35.38)	

N = number, CAR = C-reactive protein-Albumin ratio, AJCC = American Joint Committee on Cancer, NLR = neutrophil-lymphocyte ratio, PLR = platelet-lymphocyte ratio.

### Survival analysis

Among the 206 patients, during a median of 75.5 months of follow up (range: 8-136 months), local recurrence or metastatic disease after curative surgical resection was diagnosed in 55 of 141 (38.3%) patients with a low CAR and in 43 of 65 (64.6%) patients with a high CAR (P = 0.001). Regarding OS, death occurred in 27 of 141 (19.1%) patients with a low CAR and 36 of 65 (55.4%) patients with a high CAR (P = 0.000).

**Figure 2:**
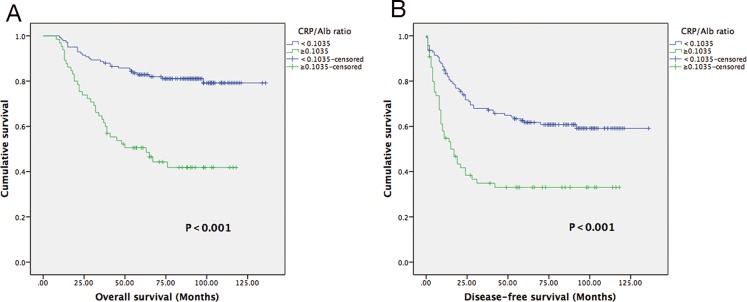
Kaplan-Meier curves showing OS **(A)** and DFS **(B)** according to the preoperative optimal value of the CAR in STS patients.

The low-CAR group exhibited a longer median overall survival and higher 5-year OS rate than the high-CAR group (115.8 vs 68.2 months, P = 0.000; 80.9% vs 44.6%, P = 0.000), respectively (Figure 2A). In multivariate analysis, a high CAR was significantly associated with decreased OS (Hazard ratios (HR): 2.47; 95% CI:1.47-4.14; P = 0.001). In addition, the tumor grade was also identified as independent prognostic factor, but the AJCC stage, Hs-mGPS, NLR and PLR were not (Table 3).

**Table 3:** Uni-and multivariate analyses for OS and DFS

	OS				DFS			
	Univariate analysis		Multivariate analysis		Univariate analysis		Multivariate analysis	
	HR(95% CI)	*p* value	HR(95% CI)	*p* value	HR(95% CI)	*p* value	HR(95% CI)	*p* value
Sex								
male	1(referent)				1(referent)			
female	1.61(0.98-2.65)	0.059			1.12(0.75-1.68)	0.582		
Age (years)								
<50	1(referent)				1(referent)		1(referent)	
≥50	1.25(0.75-2.09)	0.394			1.74(1.16-2.60)	0.007	1.72(1.12-2.64)	0.013
Tumor size								
<5cm	1(referent)				1(referent)			
≥5cm	1.42(0.85-2.36)	0.181			2.09(1.38-3.18)	0.001		
Tumor depth								
Superficial	1(referent)				1(referent)			
Deep	2.28(1.32-3.95)	0.003			2.15(1.40-3.31)	0.000		
Tumor grade								
G1	1(referent)		1(referent)		1(referent)		1(referent)	
G2	13.83(1.85-103.31)	0.010	10.93(1.45-82.16)	0.020	2.09(1.08-4.06)	0.029	1.74(0.89-3.40)	0.015
G3	44.27(6.08-322.33)	0.000	33.02(4.49-242.74)	0.001	5.10(2.69-9.69)	0.000	4.460(2.32-8.59)	0.000
AJCC stage								
IA+IB	1(referent)				1(referent)			
IIA+IIB	9.57(2.29-40.00)	0.002			2.17(1.17-4.03)	0.015		
III+IV	20.44(4.86-85.89)	0.000			5.13(2.70-9.76)	0.000		
Hs-mGPS								
0	1(referent)				1(referent)			
1	2.70(1.58-4.58)	0.000			2.10(1.38-3.18)	0.000		
2	4.96(2.02-12.18)	0.000			2.79(1.26-6.20)	0.012		
NLR								
<1.64	1(referent)				1(referent)			
≥1.64	2.20(1.23-3.94)	0.008			1.72(1.11-2.68)	0.016		
PLR								
<151.9	1(referent)				1(referent)			
≥151.9	2.22(1.33-3.71)	0.002			1.61(1.05-2.47)	0.029		
CAR								
<0.1035	1(referent)		1(referent)		1(referent)		1(referent)	
≥0.1035	3.69(2.22-6.16)	0.000	2.47(1.47-4.14)	0.001	2.44(1.62-3.66)	0.000	1.88(1.22-2.91)	0.004

HR = hazard ratio, CI = confidence interval, AJCC = American Joint Committee on Cancer, Hs-mGPS = high sensitivity modified Glasgow prognostic score, NLR = neutrophil-lymphocyte ratio, PLR = platelet-lymphocyte ratio, CAR = C-reactive protein-Albumin ratio.

The low-CAR group presented a median DFS of 63 months, whereas the high-CAR group had a median DFS of 15 months (Figure 2B). In univariate analysis, a low-CAR was significantly associated with longer DFS (HR: 2.44; 95% CI:1.62-3.66; P = 0.000), and this finding remained significant in the multivariate analysis (HR: 1.88; 95% CI:1.22-2.91; P = 0.004) that included tumor grade and age (Table 3).

**Figure 3:**
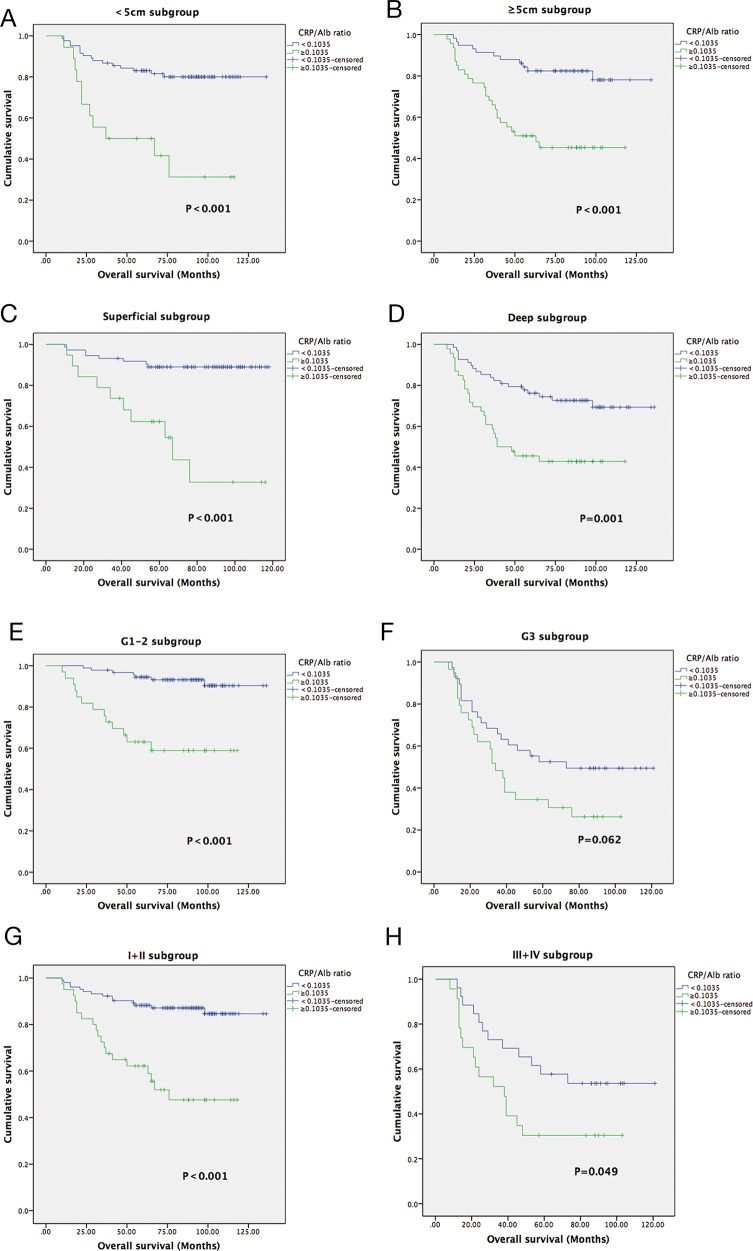
Kaplan-Meier curves showing OS according to the preoperative optimal value of the CAR in <5 cm subgroup **(A)**; ≥5 cm subgroup **(B)**;Superficial subgroup **(C)**; Deep subgroup **(D)**; G1-2 subgroup **(E)**; G3 subgroup **(F)**; I+II subgroup **(G)**; and III+IV subgroup **(H)**.

**Figure 4:**
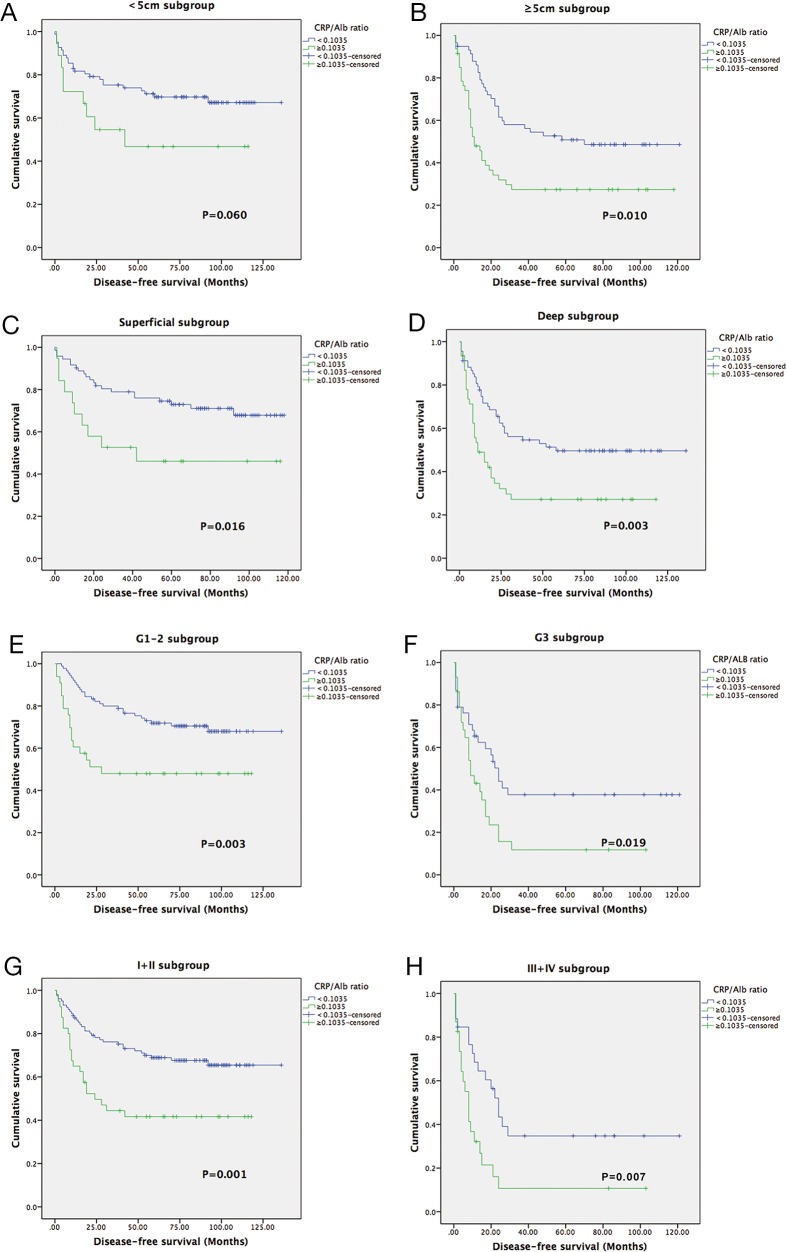
Kaplan-Meier curves showing DFS according to the preoperative optimal value of the CAR in <5 cm subgroup **(A)**; ≥5 cm subgroup **(B)**;Superficial subgroup **(C)**; Deep subgroup **(D)**; G1-2 subgroup **(E)**; G3 subgroup **(F)**; I+II subgroup (G); and III+IV subgroup **(H)**.

In individual subgroup analyses, we found that a longer OS and DFS were also observed in patients in the low-CAR group in the superficial subgroup (P<0.001 and P = 0.016), deep subgroup (P = 0.001 and P = 0.003), G1/G2 (P<0.001and P = 0.003), early stage I/II (P<0.001 and P = 0.001), and advanced stage III/IV (P = 0.049 and P = 0.007) but not in the G3 subgroup for OS (P = 0.062) and in the <5 cm subgroup for DFS (P = 0.060) (Figure 3, Figure 4).

## DISCUSSION

To the best of our knowledge, this is the first study that evaluates the prognostic value of the CAR in patients with STS. The outcomes of this retrospective study showed that the CAR was an independent prognostic factor for OS and DFS in STS. These results are consistent with previous studies identifying the CAR as a predictor of outcome in other cancers [16, 17].

In recent years, increasing evidence has demonstrated that cancer-associated inflammation plays an important role in carcinogenesis and tumor progression. It is estimated that up to 20% of cancers are initiated by chronic inflammation or persistent infections. Probable mechanisms include inflammation associated with malnutrition, immune dysfunction, up-regulation of growth factors, and angiogenesis. In addition, inflammatory cytokines produced by cancer cells (tumor necrosis factor (TNF); interleukin (IL)-1, -6, and -8; and vascular endothelial growth factor (VEGF)) also provide a favorable environment to facilitate tumor growth, invasion, and metastasis [18].

As a sensitive and reliable prognostic marker for systemic inflammation, serum CRP is synthesized mainly by hepatocytes and is predominantly under the control of IL-6 [19]. Some research has validated the prognostic value of CRP in a variety of primary malignancies [20]. The potential mechanisms and several possible explanations have been postulated. First, tumor growth and invasion could promote inflammation and hence increase CRP levels [21]. Second, CRP could represent a modulator of the immunological system of the host to tumor antigens [22]. Third, tumor cell necrosis could increase the production of CRP or positively upregulate aforementioned inflammatory cytokines to increase CRP levels [23]. Szkandera J, et al. reviewed 304 patients and found that elevated CRP level is associated with poor survival in patients with STS [24]. This conclusion was corroborated by a meta-analysis [25].

As a chronic phase protein, serum Alb is currently considered an indicator of a patient's nutritional and inflammatory status. Low serum Alb levels caused by poor performance, weight loss, and malnutrition have further been recognized as an indicator of the consuming nature of the neoplasm and are associated with poor prognosis in cohorts of different malignancies, including STS [26]. This finding is explained by the fact that hypoalbuminemia can cause a series of detrimental clinical effects, including edema, impaired organ function and microenvironmental variations, thereby reducing treatment response and leading to tumor progression. In addition, concomitant nutritional decline reduces a patient's capacity to endure aggressive cancer therapy [27].

The CAR, a novel continuous marker that indicates the interactive relationship between systemic inflammatory responses and dystrophy, was first reported to identify patients with acute illness in an acute medical ward [28]. Recently, several studies have demonstrated that the CAR is a promising prognostic factor in cancer [12–15, 17]. These achievements greatly piqued our interest in identifying the prognostic value of the CAR in STS.

In our study of 206 STS patients, we identified significant relationships among CRP, Alb and OS, which might suggest that a comprehensive evaluation of these two parameters could provide a more advisable prognostic estimate. In addition, according to the ROC curve, the AUC value of the CAR was higher than that of the NLR and PLR and, equals to that of the Hs-mGPS. The optimal CAR cutoff value of 0.1035 was applied to predict OS. This result presented a more convincing conclusion that the CAR exhibited comparable prognostic ability with other inflammation-based prognostic scores and was even stronger than NLR and PLR. Of note, the AUCs of the CAR and Hs-mGPS, which are determined using CRP and Alb values, are larger than those of the NLR and PLR, which are a white blood cell-based prognostic score. This finding indicates that the CRP-based prognostic score is preferable to the white blood cell-based prognostic score. However, Hs-mGPS may overestimate (a lower Alb level) or underestimate (a lower CRP level) the inflammatory level because it separates the values of CRP and Alb and evaluates them independently based on categorization. In contrast, the CAR reduces these potentials and may be superior to the Hs-mGPS due to the presence of a continuous variable.

More importantly, we found that an increased CAR was significantly associated with larger tumor size, higher tumor grade and more advanced AJCC stage, suggesting that increased CAR may correlate with a more aggressive tumor behavior in STS patients. In ovarian cancer, Liu Y *et al*. also found that an elevated CAR was associated with advanced stage, residual tumor, ascites and elevated serum carbohydrate antigen(CA)-125 level [17]. They demonstrated that a high CAR significantly paralleled tumor progression. Their conclusion was consistent with our study. And also, these results indicated that the CAR may give prognostic information and improve patient risk stratification for identifying those who are likely to benefit from adjuvant therapy, and by extension, neoadjuvant therapy as well.

On univariate analysis, all inflammation-based prognostic scores exhibited statistical significance regarding OS and DFS. However, after excluding the confounding factors using a Cox regression model of multivariate analysis, we found that only the CAR rather than other inflammation-based prognostic scores was independently associated with OS and DFS. This finding suggested that the CAR has a substantial impact on patient outcome. Surprisingly,AJCC stage is no longer an independent predictor of OS and DFS, and this result is in contrast with a previous large-scale study [29]. The reason for this difference is that the AJCC stage system may have some flaws due to the limitation of subjective evaluation criteria and lack of effective prognostic factors. The recently released eighth edition of the AJCC TNM staging manual for STS represents an unprecedented change in risk stratification of patients with sarcomas [30]. The manual define T-stage categories for the different primary tumor sites. However, these cutoff values are still controversial. Our data suggest that the AJCC staging system may have to be modified to improve risk stratification according to the results of new prognostic indicators, and the CAR is a potential prognostic factor for STS.

Of note, these findings may provide a new and valuable clue for individualized treatment and surveillance of STS patients. Patients with an increased CAR may require more frequent follow-up and intense therapy. Moreover, as an easily detectable biomarker, the CAR has the advantage of being simple to measure and standardized without any other complicated expenditure, thus offering reduced cost and increased convenience for prognostication.

There are a few potential limitations associated with this study. First, our study was a retrospective design involving a single-center with a small sample size; thus, clinical and survival comparisons might be influenced by selection bias. Second, heterogeneity is noted in the treatments for STS patients after surgery. In addition, other nutritional indexes, such as BMI or prognostic nutritional index (PNI), which were recognized as prognostic factors, are lacking in our retrospective data. Thus, future studies, especially prospective multicenter clinical trials with a large cohort, are required to solve these problems.

## MATERIALS AND METHODS

### Patients

Between November 2005 and August 2013, all STS patients who underwent extensive and radical resection at Sun Yat-sen University Cancer Center (SYSUCC) were carefully retrospectively reviewed. The following eligibility criteria were used: (1) patients who were pathologically diagnosed as STS; (2) patients who survived at least 30 days postoperatively; (3) patients who did not receive any neoadjuvant therapy before serum collection; and (4) patients whose preoperative laboratory data were available. Patients with synchronous cancer, acute infection or chronic inflammatory diseases were excluded. Finally, a total of 206 patients were retrospectively enrolled in this study.

This study was approved by the Institutional Review Board of Sun Yat-sen University Cancer Center. All patients provided written informed consent for their information to be stored and used in the hospital database.

### Clinical data collection

The laboratory data, including laboratory counts of neutrophils, lymphocytes and platelets as well as CRP and Alb levels, were obtained by preoperative exploration 1–7 days before surgery. Clinical data, such as age at diagnosis, gender and histopathological diagnosis were retrospectively collected from the patients’ histories. The stage was classified according to the AJCC 7th Edition [31], and tumors were graded according to the French Federation of Cancer Centers Sarcoma Group (FNCLCC) grading system [32]. The Hs-mGPS, which was reported in a previous study [7], was calculated using the CRP and Alb values as follows. Patients with both hypoalbuminemia (<3.5 g/dl) and an elevated CRP level (>0.3 mg/dl) were given a score of 2. Those who had only an elevated CRP level were assigned a score of 1. The remaining patients were assigned a score of 0. The NLR, PLR and CAR were calculated based on the following formulas:

NLR = Neutrophil count/lymphocyte count;

PLR = Platelet count/lymphocyte count; and

CAR = Serum CRP level/serum Alb level.

The authenticity of this article was validated by uploading the key raw data to the Research Data Deposit public platform (www.researchdata.org.cn) with the approval RDD number of RDDA2017000180.

### Patient follow-up

Follow-up programs were provided by the independent follow-up program department in Sun Yat-sen University at regular intervals. The final survival follow-up time was considered the latest follow-up date of this study (May 01, 2017) or death. OS, which was defined as the main endpoint, was calculated from the date of the operation to the date of death from any cause or last follow-up. The secondary endpoint was DFS, which was determined from the date of curative resection to the date of tumor recurrence or distant metastasis.

### Statistical analysis

The data are presented as the number (%), and comparisons between groups were performed using the chi-square (χ2) test. Pearson correlation was conducted to evaluate the relationship between serum CRP and Alb with OS. The optimal cut-off points for NLR, PLR and CAR were determined by ROC analysis, and the AUC were calculated. Survival curves were analyzed according to the Kaplan–Meier method, and differences between survival rates among different groups were compared using the log-rank test. Prognostic variables associated with OS and DFS that were significant in univariate analyses were selected for multivariate Cox proportional hazard model analyses using the forward stepwise method. HR estimated from the Cox analysis were reported as relative risks with corresponding 95% CI. The differences were considered statistically significant when P <0.05. All analyses were performed using SPSS version 20.0 (SPSS Inc., Chicago, IL, USA.).

## SUPPLEMENTARY MATERIALS FIGURES AND TABLES


