# Supplementary material for: Prognostic value of the C-reactive protein/Albumin Ratio (CAR) in patients with operable soft tissue sarcoma
Source: Oncotarget. 2017 Sep 18;8(58):98135–47. doi: 10.18632/oncotarget.20990 (PMC5716719; doi:10.18632/oncotarget.20990)
Supplement: Supplementary file 1 [file oncotarget-08-98135-s001.pdf]

Prognostic value of the C-reactive protein/Albumin Ratio (CAR) in patients with operable soft tissue sarcoma

SUPPLEMENTARY MATERIALS

Supplementary Table 1: AUC of the inflammation-based prognostic scores

	AUC	95%CI	*p* value
CAR	0.662	0.577-0.748	0.000
Hs-mGPS	0.662	0.579-0.748	0.000
NLR	0.612	0.527-0.697	0.011
PLR	0.624	0.535-0.713	0.005

AUC = area under curve, CI = confidence interval, CAR = C-reactive protein-Albumin ratio, NLR = neutrophil-lymphocyte ratio, PLR = platelet-lymphocyte ratio.

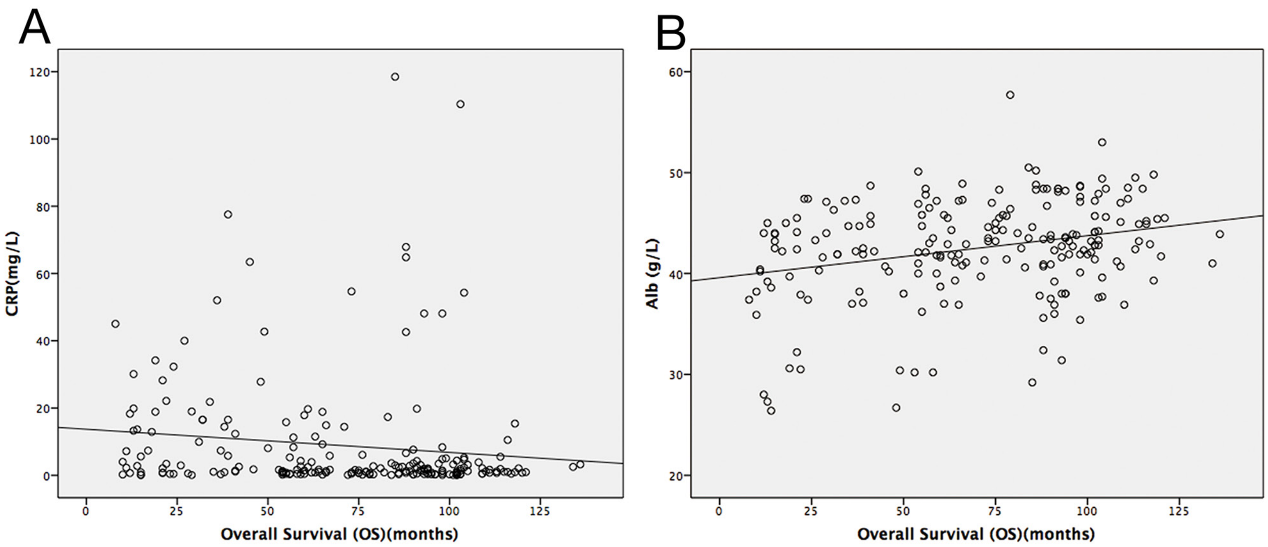

Supplementary Figure 1: The relationship of serum CRP (**A**), Alb (**B**) with OS.
